# Supplementary material for: NMR analysis of nucleotide π-stacking in prebiotically relevant crowded environment
Source: Commun Chem. 2020 Apr 30;3:51. doi: 10.1038/s42004-020-0300-7 (PMC9814533; doi:10.1038/s42004-020-0300-7)
Supplement: Supplementary file 1 — Supplementary Information [file 42004_2020_300_MOESM1_ESM.pdf]

NMR analysis of nucleotide π-stacking in prebiotically relevant crowded environment

Authors: Niraja V. Bapat[#a], Harshad Paithankar[#b], Jeetender Chugh*[ab], Sudha Rajamani*[a]

Affiliations: [a]Department of Biology, Indian Institute of Science Education and Research (IISER), Dr. Homi Bhabha road, Pashan, Pune-411008, India

[b]Department of Chemistry, Indian Institute of Science Education and Research (IISER), Dr. Homi Bhabha road, Pashan, Pune-411008, India

# Equal contribution

* Corresponding authors

Lead contact:

Sudha Rajamani, PhD: srajamani@iiserpune.ac.in

Running title: Prebiotic implications of nucleotide π-stacking in a crowded environment

Supplementary Information

Supplementary Table 1. Diffusion constants for the nucleotides in the absence and presence of PEG

Concentration of Nucleotide (in mM)	Diffusion constant ($1*E^{-10}$) (m^2/s) without PEG	Diffusion constant ($1*E^{-10}$) (m^2/s) with PEG
**5'-AMP**		
10	$5.08E^{-10} \pm 0.77E^{-12}$	$1.75E^{-10} \pm 1.18E^{-12}$
50	$4.84E^{-10} \pm 1.31E^{-12}$	$1.62E^{-10} \pm 0.68E^{-12}$
100	$4.51E^{-10} \pm 2.23E^{-12}$	$1.64E^{-10} \pm 0.43E^{-12}$
**5'-GMP**		
10	$4.98\ E^{-10} \pm 2.18E^{-12}$	$1.93E^{-10} \pm 2.20E^{-12}$
50	$4.57E^{-10} \pm 2.42E^{-12}$	$1.60E^{-10} \pm 0.94E^{-12}$
100	$4.29E^{-10} \pm 0.60E^{-12}$	$1.44E^{-10} \pm 0.45E^{-12}$
**5'-CMP**		
10	$5.53E^{-10} \pm 1.84E^{-12}$	$1.44E^{-10} \pm 5.05E^{-12}$
50	$5.29E^{-10} \pm 0.62E^{-12}$	$1.98E^{-10} \pm 1.02E^{-12}$
100	$5.06E^{-10} \pm 1.45E^{-12}$	$1.96E^{-10} \pm 0.40E^{-12}$
**5'-UMP**		
10	$5.57E^{-10} \pm 4.22E^{-12}$	$1.87E^{-10} \pm 5.03E^{-12}$
50	$5.38E^{-10} \pm 1.59E^{-12}$	$1.99E^{-10} \pm 2.15E^{-12}$
100	$5.03E^{-10} \pm 1.29E^{-12}$	$1.92E^{-10} \pm 0.18E^{-12}$

Supplementary Table 2: T_1 relaxation time data for different nucleotides in the absence and presence of 18% PEG 8000 or dextran

Concentration of Nucleotide (in mM)	Without co-solute		With PEG 8000		With Dextran	
	T_1 (ms) at 10 °C	T_1 (ms) at 25 °C	T_1 (ms) at 10 °C	T_1 (ms) at 25 °C	T_1 (ms) at 10 °C	T_1 (ms) at 25 °C
**5'-AMP**						
10	468.48 ± 11.92	580.62 ± 14.94	271.54 ± 13.63	338.48 ± 10.87	325.23 ± 2.80	388.17 ± 3.26
40	361.69 ± 7.63	510.30 ± 8.26	287.24 ± 6.15	338.86 ± 3.57	315.04 ± 4.82	349.31 ± 6.73
100	326.10 ± 6.48	431.74 ± 4.39	271.16 ± 6.95	309.71 ± 12.16	309.92 ± 3.46	318.77 ± 7.00
**5'-GMP**						
10	383.65 ± 22.77	469.11 ± 3.26	317.14 ± 15.44	280.72 ± 21.94	277.39 ± 22.21	284.58 ± 17.78
40	340.42 ± 4.87	429.74 ± 15.62	244.62 ± 7.48	323.08 ± 4.67	303.84 ± 7.87	332.68 ± 8.33
100	297.11 ± 2.90	369.39 ± 2.98	253.91 ± 6.78	315.45 ± 7.27	294.12 ± 5.58	310.94 ± 10.78
**5'-CMP**						
10	391.23 ± 11.70	531.67 ± 5.75	289.84 ± 6.20	342.47 ± 18.37	283.59 ± 2.77	342.78 ± 7.71
40	380.12 ± 3.01	520.03 ± 6.81	284.39 ± 1.19	365.06 ± 6.87	303.08 ± 16.10	341.47 ± 2.71
100	361.04 ± 2.90	493.06 ± 4.55	271.92 ± 1.97	351.37 ± 4.49	275.15 ± 2.69	331.93 ± 1.85
**5'-UMP**						
10	384.18 ± 5.39	547.65 ± 6.17	272.89 ± 6.57	385.46 ± 15.27	281.96 ± 3.90	350.84 ± 4.60
40	378.79 ± 2.81	522.71 ± 4.02	292.04 ± 6.19	369.22 ± 10.55	275.56 ± 2.93	349.10 ± 2.54
100	368.43 ± 2.04	506.53 ± 5.27	280.27 ± 1.94	370.59 ± 5.05	267.57 ± 1.19	341.46 ± 3.60

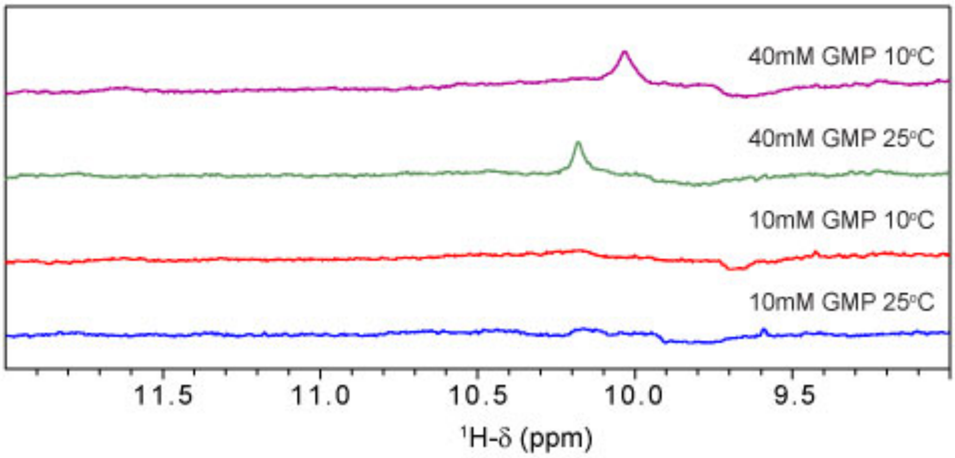

Supplementary Figure 1: Presence of G-quadruplex structure in 40 mM 5'-GMP concentration as indicated by the peak near 10 ppm in ^{1}H NMR

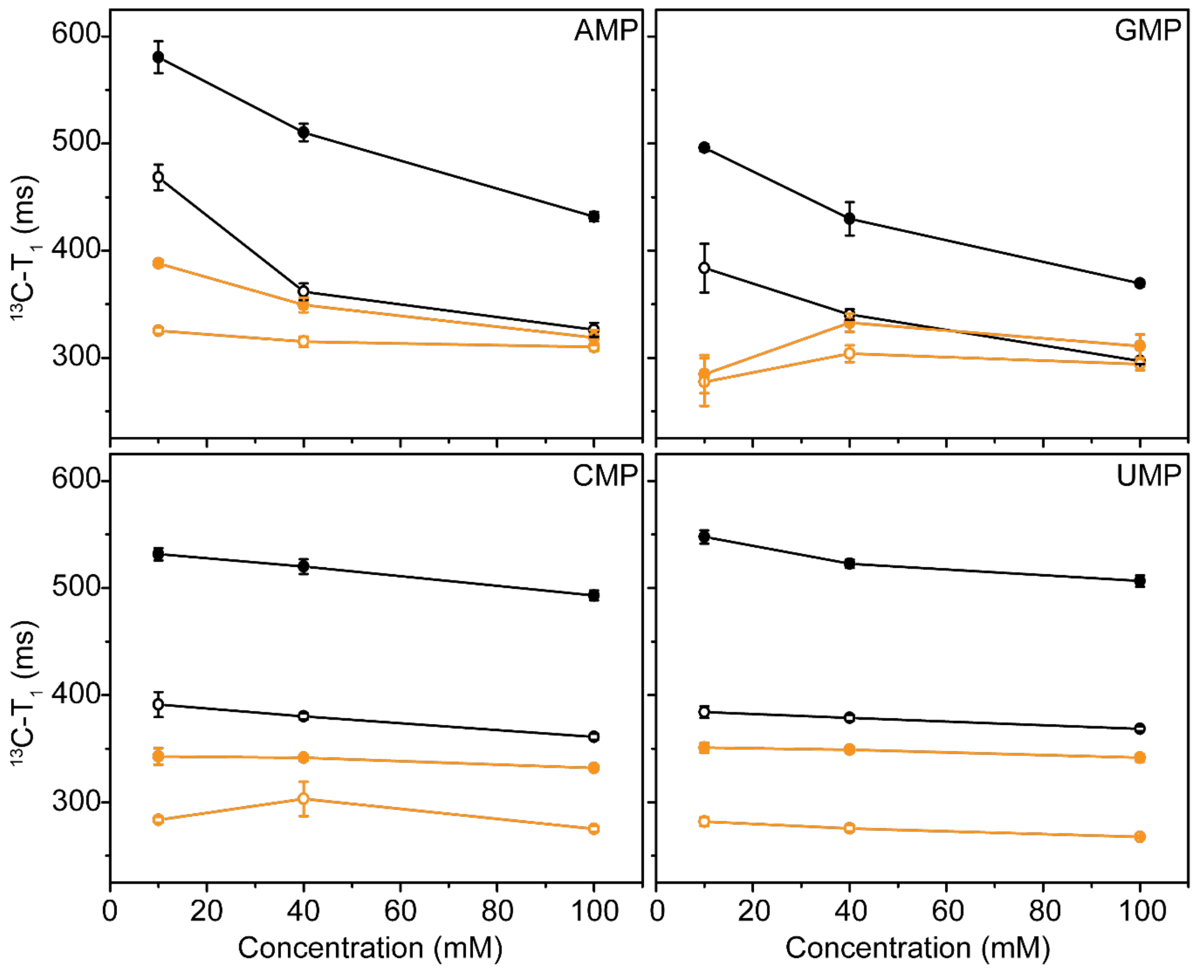

Supplementary Figure 2: 13C-T1 relaxation time data of nucleotides at different concentrations. The data was recorded in absence of crowding agent (Black) and in presence of Dextran (orange) at 25 °C (filled circles) and 10 °C (empty circles) for all four nucleotides as labeled in the top right corner of each of the plot. The error bars are obtained as a fit error to the mono-exponential decay equation 5, using repeat point and Monte-Carlo simulations.

Supplementary Table 3. Experimental parameters, Δ and δ, that are used to record DOSY data for nucleotides at different concentrations

Concentration of Nucleotide (in mM)	Without PEG		With PEG 8000	
	Δ (ms)	δ (ms)	Δ (ms)	δ (ms)
**5'-AMP**				
10	60	2.5	60	4.5
50	60	2.6	60	4.5
100	60	2.8	60	4.8
**5'-GMP**				
10	60	2.5	60	4.4
50	60	2.6	60	4.4
100	60	2.6	60	4.4
**5'-CMP**				
10	60	2.5	60	3.8
50	60	2.6	60	4.0
100	60	2.8	60	4.0
**5'-UMP**				
10	60	2.5	90	3.4
50	60	2.6	65	3.8
100	60	2.7	60	4.0
